# Elective cholecystectomy benchmarks: nationwide registry study

**DOI:** 10.1093/bjsopen/zrag175

**Published:** 2026-10-13

**Authors:** Ebba Kihlstedt Pasquier, Ellen Andersson, Johanna Österberg, Per Sandström, Bergthor Björnsson

**Affiliations:** Department of Biomedical and Clinical Sciences, Linköping University, Linköping, Sweden; Clinical Department of Surgery in Linköping, Region Östergötland, Linköping, Sweden; Department of Biomedical and Clinical Sciences, Linköping University, Linköping, Sweden; Clinical Department of Surgery in Norrköping, Region Östergötland, Norrköping, Sweden; Department of Clinical Science and Education, Södersjukhuset, Karolinska Institutet, Stockholm, Sweden; Department of Surgery, Mora Lasarett, Mora, Sweden; Department of Biomedical and Clinical Sciences, Linköping University, Linköping, Sweden; Clinical Department of Surgery in Linköping, Region Östergötland, Linköping, Sweden; Department of Biomedical and Clinical Sciences, Linköping University, Linköping, Sweden; Clinical Department of Surgery in Linköping, Region Östergötland, Linköping, Sweden

**Keywords:** benchmarking, laparoscopy, surgical outcomes, quality improvement, GallRiks registry

## Abstract

**Background:**

Laparoscopic cholecystectomy is the treatment of choice for symptomatic gallstone disease. The aim of this study was to establish clinically relevant benchmarks for elective laparoscopic cholecystectomy (ELC) to guide quality initiatives and support improvements in patient outcomes.

**Method:**

The Swedish Registry for Gallstone Surgery and Endoscopic Retrograde Cholangiopancreatography provided data on patients treated with primary ELC for a benign indication between 2015 and 2023. Exclusion criteria were common bile duct stones or stents detected before surgery, suspicion of bile duct obstruction, abnormal or unperformed intraoperative cholangiogram, previous major upper abdominal surgery, concomitant surgical procedures, and robotic surgery. The Achievable Benchmark of Care methodology was used to develop intra- and postoperative benchmarks, with severe postoperative complications (Clavien–Dindo ≥ III) representing the primary outcome. During surgery, serious adverse events were defined as intestinal perforation, bile leakage, or bleeding requiring transfusion or conversion to open surgery. Postoperative outcomes were assessed up to 30 days after surgery. Logistic regression identified risk factors for severe postoperative complications.

**Results:**

Overall, 48 564 patients undergoing ELCs across 81 surgical facilities were included. Among these, 69% were women. Median age was 50 (range 18–95) years and 91.4% were classified as American Society of Anesthesiologists classification ≤ II. Ten benchmarks were developed, capturing either intraoperative events (3) or postoperative complications (7). During surgery, 0.5% of the total cohort experienced a serious adverse event (median 0.3%, interquartile range 0.2–0.7% between facilities)—benchmark was 0.2%. Severe postoperative complications occurred in 1.7% of patients (median 1.6%, interquartile range 1.0–2.2% between facilities)—benchmark was 1.0%. Postoperative mortality was low (7 deaths, < 0.1%). At multivariable analysis, American Society of Anesthesiologists classification ≥ III (odds ratio 1.35, 95% confidence interval 1.05 to 1.72; *P* = 0.018), previous abdominal surgery (odds ratio 2.15, 1.24 to 3.75; *P* = 0.007), and intraoperative assessment of an altered gallbladder morphology (odds ratio 1.24, 1.01 to 1.52; *P* = 0.044) were independently associated with severe postoperative complications.

**Conclusion:**

This study provides benchmarks for elective laparoscopic cholecystectomy. When compared with real-world performance, these benchmarks highlight potential areas for quality improvement.

## Teaser

This nationwide registry study establishes achievable benchmarks for elective laparoscopic cholecystectomy based on outcomes from top-performing surgical facilities. Ten indicators reflecting intraoperative and postoperative performance were assessed, and risk-adjusted benchmarks were defined for severe postoperative complications. Together, these benchmarks provide reference standards for comparison and may guide surgical quality improvement.

## Introduction

Laparoscopic cholecystectomy is among the most common surgical procedures globally^1^. Although considered routine surgery, severe complications occur and variations in perioperative outcomes exist^2^. Given the high procedural volume, even small differences in performance may have substantial implications for patient safety and healthcare quality. Despite numerous studies on different outcomes, standardized benchmarks are lacking^3–5^.

Conventional outcome reporting typically describes average performance and therefore provides limited guidance regarding what outcomes are realistically achievable in contemporary clinical practice. Benchmarking is a methodological approach aimed at defining standards of excellence by identifying best attainable performance, which may serve as targets for performance evaluation and quality improvement. Recently, it has gained increasing recognition within the field of surgery^6^. In particular, the Achievable Benchmark of Care (ABC™) methodology offers a possibility to evaluate outcomes in unselected patient populations and incorporates statistical adjustment for case volume, thereby allowing inclusion of low-volume facilities while reducing their influence^7,8^. The resulting benchmarks reflect attainable results within real-world healthcare systems.^9^

The aim of this study was to define benchmarks for routine elective laparoscopic cholecystectomy (ELC), applying the ABC™ benchmarking methodology to identify best attainable peri- and postoperative outcomes. Specifically, the primary aim was to benchmark severe postoperative complications, defined as Clavien–Dindo (CD) ≥ III, with adjustment for preoperative risk factors^10^. Benchmarking other intra- or postoperative outcomes was considered a secondary aim.

## Methods

### Study design and data source

The study cohort was retrospectively gathered through the prospectively maintained, national Swedish Registry for Gallstone Surgery and Endoscopic Retrograde Cholangiopancreatography (GallRiks), which has demonstrated good coverage (95%) and high internal validity (> 97%) in previous studies^11,12^. Surgical data were registered by surgeons and endoscopists, and follow-up data were submitted by local coordinators. The study was approved by the Swedish Ethical Review Authority (Dnr 2022-04311-0), with a subsequent amendment to include an additional 2 years of data (Dnr 2024-06020-02), and was preregistered (registration number: NCT06692517, www.clinicaltrials.gov). According to the Swedish regulations, individual patient informed consent was not required, as patients were informed of their right to opt out of the registry. The manuscript was prepared in accordance with the STROBE guidelines^13^ (*Appendix S1*).

### Eligibility

All patients aged ≥ 18 years undergoing primary ELC for biliary colic, gallbladder polyps, or gallstone-related complications (previous cholecystitis, pancreatitis, or common bile duct (CBD) stone/elevated bilirubin) between January 2015 and December 2023 were eligible for inclusion. Patients with CBD stones or stents detected before surgery, suspicion of bile duct obstruction due to jaundice, elevated total bilirubin > 30 µmol/l, or abnormal or unperformed intraoperative cholangiograms (IOCs) were excluded to avoid influence of other procedures (for example endoscopic retrograde cholangiopancreatography (ERCP) or transcystic CBD stone extraction) on intra- and postoperative results. To minimize differences in case-mix between surgical facilities, patients undergoing concomitant procedures or robotic surgery were excluded, as were those with a history of major upper abdominal surgery. Robotic cholecystectomy was rarely performed during the study period and has remained uncommon in Sweden thereafter. Excluded procedures comprised previous liver, pancreatic, oesophageal, gastric, duodenal, and splenic surgery, including bariatric procedures, major resections, reconstructive procedures, and surgery for traumatic injuries. All other previous abdominal procedures were retained and are hereafter referred to as previous abdominal procedures.

### Surgical technique

In Sweden, ELC is generally performed using a four-trocar technique, with the dissection usually starting at the cystic pedicle and ending at the gallbladder fundus. IOC is performed in the vast majority of cases, aiming at early detection of bile duct stones and iatrogenic bile duct injuries^14,15^. Preoperative antibiotic prophylaxis is usually not administered^16^. Indications for thromboprophylaxis, as well as the choice of agent and duration of treatment, are determined according to clinical practice at each participating facility.

### Achievable benchmarks of care

The ABC™^7,8^, previously used to establish benchmarks for other surgical interventions including liver and pancreatic resections^9,17,18^, was applied to generate reference values reflecting the best achievable outcomes in ELC. The values, or benchmarks, represent the average performance of the top-performing surgical facilities. To promote consistency in performance, Bayesian adjustment was used to reduce the influence of low-volume facilities. The adjusted performance is calculated by dividing the number of events plus one, by the surgical volume plus two. Facilities are ranked according to the adjusted performance, and for each variable of interest the best-performing centres are sequentially included until the benchmark cohort comprises at least 10% of all procedures in the study cohort. The average of this cohort constitutes the benchmark. An example of the benchmark calculation procedure can be found in *Tables S1, S2*. For continuous variables, surgical facilities’ median values were calculated, and the benchmark was established as the 10th percentile of those median values.

### Benchmarks

Clinically relevant intra- and postoperative outcome measures were selected for benchmarking. In particular, three intraoperative outcome measures were assessed: conversion to open surgery, severe adverse events (defined as intestinal perforation, bile leakage, or bleeding requiring conversion to open surgery or blood transfusion), and intra-abdominal drain placement. Seven postoperative outcome measures were assessed: overall postoperative complications, bile leakage, reoperation, readmission, length of hospital stay (LOS), severe postoperative complications (defined as CD ≥ III), and a composite benchmark termed postoperative intervention. The latter was defined as blood transfusion, percutaneous drainage, ERCP, or reoperation. The primary outcome was the rate of severe postoperative complications, whereas all other outcomes were considered secondary. Postoperative outcomes were recorded up to 30 days after surgery. A questionnaire regarding perioperative routines was prepared for a surgical facility consistently contributing to benchmarks, including the severe complications benchmark.

Complication outcomes were defined at the patient level. Patients were counted once for each outcome, regardless of the number of registered complications. For severe complications, the highest CD grade per patient was used when multiple complications were recorded. For the specific complication of bile leakage, all patients with a registered bile leak were included regardless of CD grade.

A CD classification variable, representing the highest CD grade among all procedure-related complications, was constructed from predefined registry variables on postoperative treatment and supplemented by free-text entries^10,19^. Existing CD grades recorded in GallRiks (introduced in 2021) were used when available. For complications without a predefined CD grade, free-text entries and registered interventions were reviewed and assigned a CD grade based on the treatment required or the most likely treatment based on the available clinical information (*Appendix S2*).

### Risk adjustment

The benchmark for severe postoperative complications was adjusted for preoperative risk factors identified in multivariable logistic regression analysis by calculating separate benchmarks for the applicable risk groups. Risk adjustment was intentionally applied only to the primary outcome, as this endpoint represents the most clinically relevant composite measure of adverse outcome and occurs with sufficient frequency to allow robust modelling. Selected clinically relevant risk factors recorded in GallRiks included age, sex, body mass index, cardiovascular disease, asthma or chronic obstructive pulmonary disease, diabetes (lifestyle- or pharmacologically treated), active smoking, increased risk of bleeding (von Willebrand disease, haemophilia, ongoing anticoagulant therapy, or international normalized ratio > 1.5), immunosuppression (ongoing immunosuppressive therapy), previous abdominal procedures considered to affect the operation, and American Society of Anesthesiologists (ASA) physical status classification.

Indication for surgery was also considered clinically relevant, as previous inflammation may result in adhesions and tissue alterations. Accordingly, patients undergoing ELC for non-inflammatory indications (biliary colic and gallbladder polyp) were segregated from those with gallstone-related complications. Gallbladders macroscopically assessed by the surgeon during surgery as having an altered gallbladder morphology (including inflammation, suspected malignancy, or other macroscopic abnormality) were also analysed, as the presence of inflammatory or fibrous tissue influences surgical complexity. Gallbladder polyps were not included in this category.

Data on comorbidities, previous abdominal procedures, and readmissions were introduced to the registry in September 2016 and were therefore analysed only for procedures performed from this date onwards.

### Mitigation of bias

To minimize reporting bias, surgical facilities with no registered severe intraoperative adverse events, no registered postoperative overall complications, or no severe postoperative complications were excluded from being part of the top-performing population contributing to the respective benchmarks. Furthermore, facilities were excluded if they had ≥ 5% of missing data in the outcome variable of interest. In particular, when investigating the specific postoperative complications of bile leakage, reoperation, and the composite variable postoperative intervention, facilities with ≥ 5% missing data in the overall complication variable were excluded. For the severe complication benchmark, facilities with ≥ 5% missing data in that specific variable, or ≥ 5% missing data in the overall complication variable, were excluded from benchmark contribution. The rationale for this mitigation of possible bias was to ensure that the resulting benchmarks would be both reliable and achievable.

### Statistical analysis

Discrete variables are presented as count and percentage, and continuous variables are presented as medians with interquartile ranges (i.q.r.). Between-group differences in categorical variables were evaluated using the χ^2^ test, and continuous variables were compared using the Mann–Whitney *U* test. Univariable and multivariable binary logistic regression analyses were performed to identify preoperative risk factors for severe complications, providing odds ratios (ORs) with 95% confidence intervals (c.i.). ASA class was dichotomized into low risk (ASA ≤ II) and high risk (ASA ≥ III) to ensure statistical power. Previous abdominal surgery became an obligatory variable in the registry in September 2016, when comorbidity variables were also introduced. Univariable analyses of these variables were therefore restricted to 2016–2023. Multivariable analysis using the direct approach was performed to adjust for confounders. Variables were considered for inclusion based on clinical relevance and the results of the univariable analysis, with *P* < 0.05 indicating statistical significance; age and sex were retained regardless of statistical significance. An additional multivariable analysis was performed to assess the association between intraoperative surgeon assessment of altered gallbladder morphology and severe postoperative complications. Complementary benchmarks of severe postoperative complications were established in potential low-risk cohorts based on the absence of previous gallstone complication or factors independently associated with severe postoperative complications at multivariable analysis. Missing values were handled by complete-case analysis in multivariable models. No imputation was performed. Statistical significance was defined as *P* < 0.05. Statistical analyses were performed using IBM SPSS Statistics^®^ 29.0.2.0 (IBM, Armonk, NY, USA).

## Results

Between 2015 and 2023, 48 564 ELCs met the inclusion criteria across 81 surgical facilities (*Fig. 1*). Surgical volume varied substantially between facilities, whereas the distribution of ASA grade was similar, with most procedures performed in patients with ASA ≤ II (*Fig. S1*).

**Figure 1:**
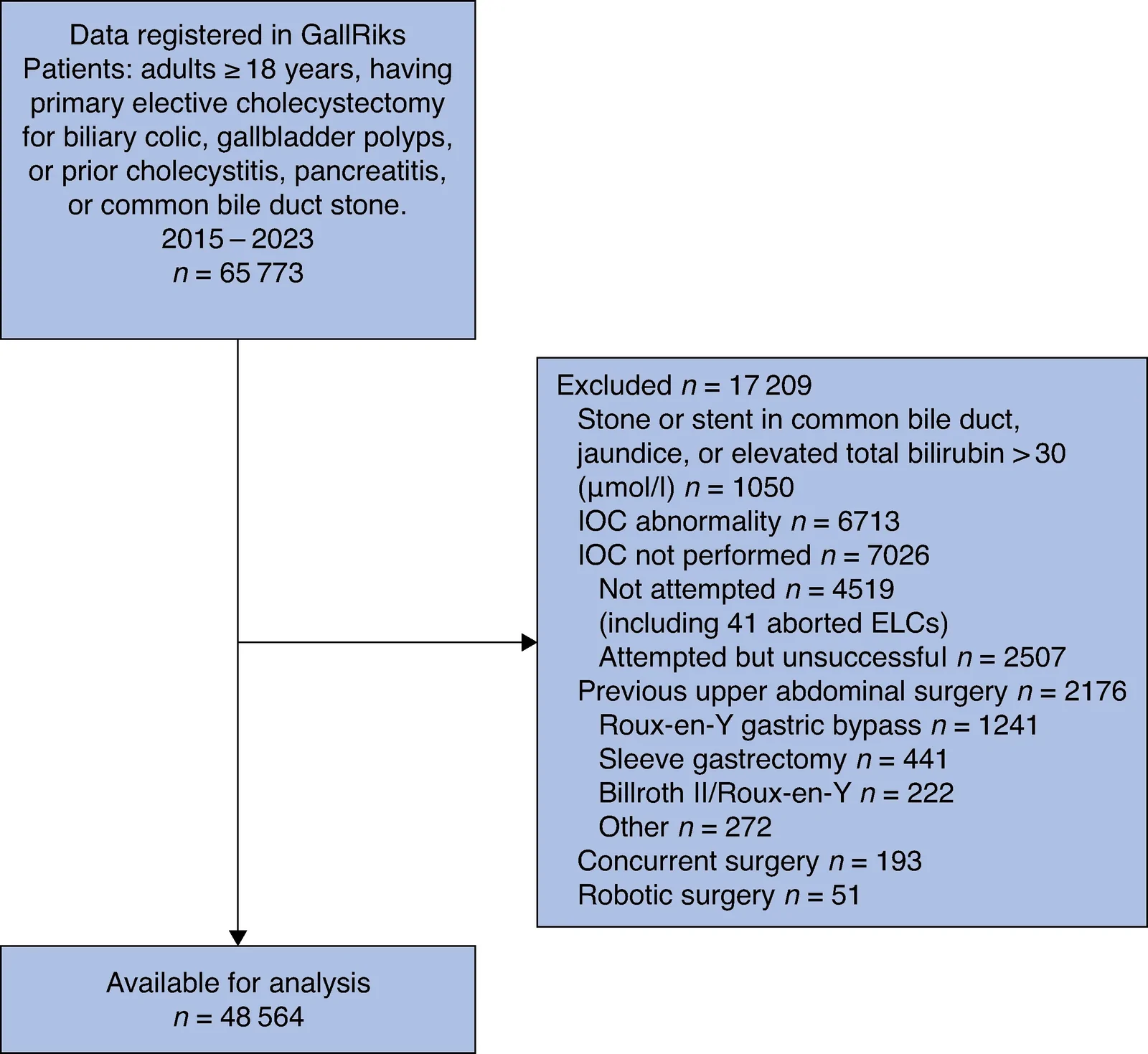
Flowchart of inclusion GallRiks, Swedish Registry for Gallstone Surgery and Endoscopic Retrograde Cholangiopancreatography; IOC, intraoperative cholangiography; ELC, elective laparoscopic cholecystectomy.

### General characteristics of the study cohort

The general characteristics of the study population are detailed in *Table S3*. Most patients were women (69.3%), and the median age was 50 (i.q.r. 38–63, range 18–95) years. Men were slightly older than women (median age 54 (i.q.r. 43–66) years *versus* 49 (i.q.r. 36–61) years; *P* < 0.001). Overall, the study population was healthy or had well-controlled comorbidities: 91.4% of the patients were classified as ASA ≤ II (median 91.8%, i.q.r. 87.9–96.8% across surgical facilities). Biliary colic was the most common surgical indication, in 74.8% of the patients (median 75.7%, i.q.r. 65.3–84.7% across facilities). It was especially common among women (27 163 (80.8%) *versus* 9173 men (61.5%); *P* < 0.001). Of patients with gallstone related complications, 7014 (60.1%) had experienced previous cholecystitis, 3657 (31.3%) pancreatitis, and 3836 (32.9%) previous bile duct obstruction (clinical jaundice, total bilirubin > 50 µmol/l, or verified CBD gallstone). As patients could present with more than one gallstone-related complication, these categories were not mutually exclusive; for details see *Table S3*.

### Surgery

Operative characteristics are summarized in *Table S4*. Median surgical duration was 77 (i.q.r. 58–102) minutes, with no missing data. Intraoperative gallbladder perforation occurred in 14 797 procedures (30.5%) and all spilled gallstones were retrieved in 14 067 (95.1%) of these cases. Serious intraoperative adverse events (bleeding, intestinal perforation, or bile duct injury) were recorded in 247 patients (0.5%), comprising 288 adverse events in total. Overall, 653 (1.3%) ELCs were converted to open surgery, most commonly due to adhesions, advanced chronic cholecystitis, or unclear anatomy.

Intraoperative assessment of altered gallbladder morphology was reported by the operating surgeon in 14 420 patients (29.7%) (median 27.6%, i.q.r. 22.5–34.9% across facilities) (*Table S4*), with suspected inflammation accounting for 14 034 (97.3%) of these findings. Among patients undergoing surgery for biliary colic or polyps, 7869 (21.3%) had a gallbladder during surgery assessed as macroscopically altered, compared with 6551 patients (56.1%) with gallstone complication as surgical indication (*P* < 0.001). Among those with previous cholecystitis, 5427 (77.4%) were macroscopically altered according to the surgeon’s assessment. A majority of gallbladders submitted for histopathology demonstrated inflammatory changes irrespective of the surgeon’s intraoperative assessment. Among patients with biliary colic or polyps, inflammatory changes were present in 4661 of 6026 (77.3%) macroscopically normal gallbladders and in 4660 of 5027 (92.7%) macroscopically altered gallbladders. Corresponding proportions among patients with previous gallstone related complications were 1074 of 1298 (82.7%) and 4482 of 4705 (95.3%), respectively.

### Postoperative course

Comprehensive 30-day postoperative data are presented in *Table 1*. Complications were registered in 2494 patients (5.1%), the most frequent being deep infection, bile leakage, and wound complications. Only 16 (5.3%) of the bile leakages were due to injuries to the CBD. In addition, 10 were due to injury of less than one-third of the circumference, 3 had an injury over one-third of the circumference, and 3 were due to a resection of the CBD. The remaining leakages originated from the cystic duct or liver bed and/or aberrant bile duct or had an unknown origin. Severe postoperative complications occurred in 836 patients (1.7%). Among patients with complications, 321 (12.9%) required reoperation, 243 (9.7%) underwent ERCP, 401 (16.1%) received percutaneous drainage, and 108 (4.3%) required a blood transfusion. The all-cause readmission rate was 3.2% (1287 patients). Postoperative mortality was low (7 deaths; < 0.1%).

**Table 1:** Thirty-day postoperative complications (total cohort 48 564)

	Patients, n	Percent of population	Percent of available responses	Missing, *n* (%)
Readmission*	1287	3.2%	NA	361 (0.9%)
**Complications**†	2494	5.1%	NA	444 (0.9%)
Abdominal pain	240	0.5%	9.6%	NA
Ileus or prolonged intestinal paralysis	24	0.0%	1.0%	NA
Bleeding, unspecified	142	0.3%	5.7%	NA
Abdominal or gastrointestinal bleeding	65	0.1%	2.6%	NA
Intestinal perforation	52	0.1%	2.1%	NA
Hernia with/without intestinal obstruction	30	0.1%	1.2%	NA
Bile leakage	291	0.6%	11.7%	NA
Bile duct obstruction	35	0.1%	1.4%	NA
Pancreatitis or cholangitis	98	0.2%	3.9%	NA
Abscess, deep infection	440	0.9%	17.6%	NA
Wound complication, including infection	283	0.6%	11.3%	NA
Pneumonia or urinary tract infection	130	0.3%	5.2%	NA
Cardiovascular, pulmonary or thrombotic complication	71	0.1%	2.8%	NA
Other complications, including nausea, constipation and urinary retention	405	0.8%	16.2%	NA
Not specified	188	0.4%	7.5%	NA
**Treatment of complications**				120 (4.8)
Reoperation	321	0.7%	12.9%	NA
ERCP	243	0.5%	9.7%	NA
Percutaneous drainage	401	0.8%	16.1%	NA
Transfusion	108	0.2%	4.3%	NA
Antibiotic therapy	872	1.8%	35.0%	NA
Free-text entry (symptom/treatment)‡	1094	2.3%	43.9%	NA
**Clavien–Dindo Classification**				120 (4.8%)
Complication CD I–II	1538	3.2%	64.8%	NA
Complication CD ≥ III	836	1.7%	35.2%	NA
CD IIIa	344	0.7%	14.4%	NA
CD IIIb	348	0.7%	14.7%	NA
CD IV	137	0.3%	5.8%	NA
CD V	7	0.0%	0.3%	NA

Values are *n* (%) unless otherwise stated. * Readmission was introduced in GallRiks in September 2016. Cohort size: 39 633 patients. †In case of several complications, the one corresponding to the highest CD category was recorded. For example, there were 300 bile leakages in total, but in 9 cases another complication caused a higher CD category. ‡The content may overlap with the other treatment alternatives. NA, not applicable; ERCP, endoscopic retrograde cholangiopancreatography. CD, Clavien–Dindo.

### Performance

Performance variables and benchmarks are displayed in *Table 2*. Patients contributing data on severe intraoperative adverse events and severe postoperative complications mirrored the ASA distribution of the overall study population (*Fig. S2*). Among facilities contributing to the benchmark for severe intraoperative adverse events, the proportion of patients with previous gallstone-related complications ranged from 5.0% to 42.7% (median 26.1%). The corresponding proportion for facilities contributing to the benchmark for severe postoperative complications ranged from 2.9% to 27.8% (median 19.7%). The case mix of the benchmark cohort for severe postoperative complications is illustrated in *Fig. 2*.

**Figure 2:**
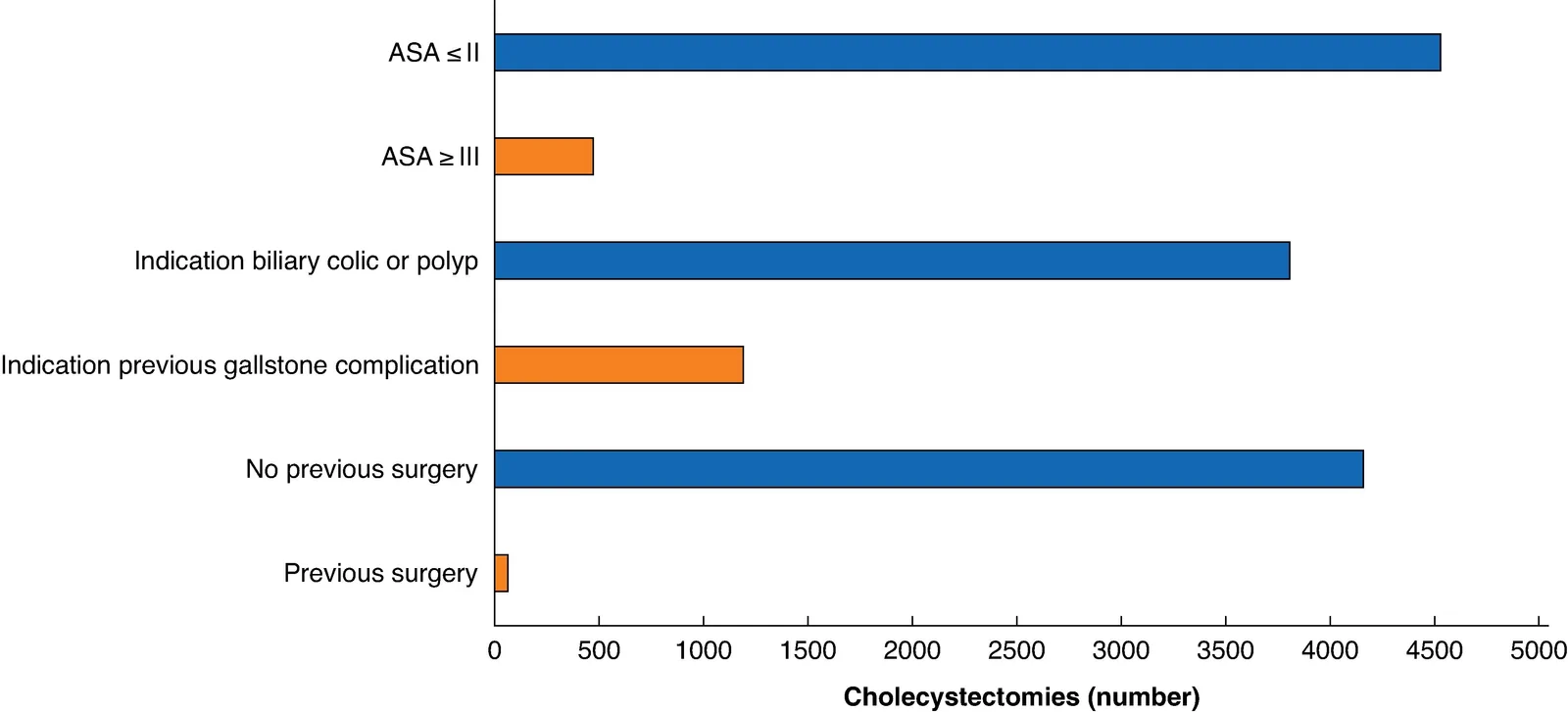
Benchmark cohort of severe postoperative complications Patient characteristics of the cohort contributing to benchmark for severe postoperative complications (4997 patients). Median age 51 (interquartile range 38–63) years. Gallstone complications: previous cholecystitis, pancreatitis, or common bile duct stone/elevated bilirubin. ASA, American Society of Anesthesiologists physical status classification.

**Table 2:** Benchmark values and interquartile performance in elective laparoscopic cholecystectomy

	Patients with events, *n* (%)	Median (IQR)	ABC™	Excluded facilities (nr)
Intraoperative drain	1578 (3.2%)	2.4% (1.2–4.0%)	0.6%	0
Conversion to open surgery	653 (1.3%)	1.0% (0.3–2.2%)	0.1%	0
Intraoperative severe adverse event*	247 (0.5%)	0.3% (0.2–0.7%)	0.2%	3*
Length of hospital stay	NA	0 days (0–1 days)	0 days	0
Any postoperative complication	2494 (5.1%)	5.1% (3.5–6.5%)	2.7%	1
Severe postoperative complication	836 (1.7%)	1.6% (1.0–2.2%)	1.0%	7‡
Postoperative intervention†	747 (1.5%)	1.5% (0.9–2.1%)	0.7%	2§
Postoperative bile leakage (any kind)	300 (0.6%)	0.5% (0.1–0.9%)	0.1%	1§
Reoperation	321 (0.7%)	0.5% (0.2–0.9%)	0.1%	0§
Readmission	1287/39 633 (3.2%)	3.2% (1.9–4.1%)	1.4%	2

Postoperative data: 0–30 days after surgery. Severe postoperative complications are defined as CD ≥ III. The combined variables adverse event (*) and postoperative intervention (†) include only one issue per patient. Excluded: number of facilities excluded from benchmark contribution due to ≥ 5% missing data. *Severe adverse events: intestinal perforation, bile leakage, and bleeding requiring transfusion or conversion to open surgery. Three facilities were excluded due to zero adverse events. †Postoperative intervention: blood transfusion, percutaneous drainage, endoscopic retrograde cholangiopancreatography, reoperation, care in intensive care unit. ‡Facilities excluded from benchmark due to ≥ 5% missing data in both complication registration and specific variable. §Facilities excluded from benchmark calculation due to ≥ 5% missing data in complication registration. IQR, interquartile range; ABC™, achievable benchmark of care; NA, not applicable; CD, Clavien–Dindo.

In total, 28 facilities contributed to at least 1 categorical benchmark, of which 13 contributed to more than 1. One facility had consistently excellent results and contributed to seven benchmarks, including that of severe postoperative complications. A questionnaire regarding their perioperative routines is presented as supplementary material, including facility case-mix (*Appendix S3*). The number of patients contributed per facility ranged from 144 to 3106 (both for the readmission benchmark). A total of 56 of 81 facilities (69%) reached the LOS benchmark of 0 days.

### Risk of severe complications

Multivariable logistic regression identified ASA ≥ III and previous intra-abdominal surgery as independent preoperative risk factors for severe postoperative complications (*Table 3*). Previous inflammation, particularly cholecystitis, was associated with intraoperative assessment of altered gallbladder morphology, but was not an independent predictor of postoperative complications after adjustment for other preoperative factors.

**Table 3:** Logistic regression analyses of risk factors for severe complications (CD ≥ III)

			Univariable analysis		Multivariable analysis	
	Patients (*n*)	CD ≥ III (*n*)	OR	P*	OR	*P*†
Age	48 564	836	1.01 (1.00, 1.01)	0.035	1.00 (0.99, 1.01)	0.806
BMI	43 950	748	1.00 (0.98, 1.02)	0.951	-	-
**Sex**						
Female	33 637	497	(reference)			
Male	14 926	339	1.09 (0.92, 1.29)	0.326	1.04 (0.86, 1.27)	0.691
**Comorbidity**						
Cardiovascular disease	6306	209	1.40 (1.14, 1.72)	0.001	-	-
Diabetes	2401	86	1.18 (0.88, 1.57)	0.267		
Asthma/COPD	2845	75	1.23 (0.91, 1.68)	0.182		
Increased risk of bleeding‡	1519	67	1.32 (0.95, 1.84)	0.095		
Immunosuppression§	723	24	1.32 (0.77, 2.24)	0.317		
Smoking	2232	38	1.24 (0.81, 1.89)	0.323		
Previous abdominal procedure	759	28	2.13 (1.22, 3.69)	0.008	2.15 (1.24, 3.75)	0.007
ASA ≥ III (reference ≤ II)	4216	172	1.49 (1.20, 1.85)	< 0.001	1.35 (1.05, 1.72)	0.018
Previous cholecystitis, cholangitis or pancreatitis¶	11 677	332	1.21 (1.02, 1.44)	0.030	1.19 (0.97, 1.46)	0.105

Values in parentheses are 95% confidence intervals. Multivariable logistic regression using the direct approach was performed to adjust for confounders. Age and sex were included based on clinical relevance together with variables with *P* < 0.05 in univariable analysis. The multivariable model was restricted to the period 2016–2023 because previous abdominal surgery was included as a potential confounder. Effect estimates were similar when including the full cohort (2015–2023). Cardiovascular disease and BMI were excluded from the final model due to conceptual redundancy with ASA class, which was considered a more comprehensive measure of comorbidity (illustrated with a hyphen in the table). *,†*P*-values were calculated using the Wald test. ‡Increased risk of bleeding: anticoagulant therapy, and haemophilia. §Immunosuppression: immunosuppressive therapy. ¶Reference: biliary colic or gallbladder polyp. CD, Clavien–Dindo; OR, odds ratio; BMI, body mass index; COPD, chronic obstructive pulmonary disease; ASA, American Society of Anesthesiologists physical status classification.

In an additional multivariable analysis, intraoperative assessment of altered gallbladder morphology was independently associated with severe postoperative complications (OR 1.24, 95% c.i. 1.01 to 1.52; *P* = 0.044) after adjustment for the same factors as in the primary multivariable analysis (age, sex, ASA grade, surgical indication, and previous abdominal surgery). The inclusion of this variable did not significantly affect the results of the primary analysis (*Table 3*).

Two exploratory subgroup analyses restricted to low-risk patients were performed: first, in patients with ASA ≤ II and normal gallbladders during surgery; and, second, in patients with ASA ≤ II undergoing ELC for biliary colic or polyp. Adjusted benchmark values for severe postoperative complications are presented in *Table 4*.

**Table 4:** Adjusted benchmarks for severe postoperative complications (CD ≥ III)

		CD ≥ III, median (IQR)	ABC™	Excluded facilities (nr)
Overall population	Total: 48 564 patients, 1.7% CD ≥ III	1.6% (1.0–2.2%)	1.0%	7*
ASA ≥ III	Total: 4216 patients, 4.1% CD ≥ III	3.4% (0.0–5.7%)	1.5%	0
Previous surgery	Total: 759 patients, 3.4% CD ≥ III	0.0% (0.0–5.0%)	1.0%	0
Intraoperative gallbladder abnormality‡	Total: 14 420 patients, 2.5% CD ≥ III	2.4% (1.3–3.9%)	1.0%	1* 1†
ASA ≤ II + no intraoperative abnormality‡	Total: 31 621 patients, 1.2% CD ≥ III	1.2% (0.5–1.6%)	0.6%	6* 2†
ASA ≤ II + indication biliary colic or polyp	Total: 34 495 patients, 1.3% CD ≥ III	1.1% (0.6–1.9%)	0.7%	10* 2†

The cohorts shown above were associated with a higher risk of severe postoperative complications, whereas the last two cohorts comprised patients with lower-risk profiles. Excluded: number of facilities excluded from benchmark contribution due to *≥ 5% missing data (complication registration or CD variable) or †zero severe postoperative complications. ‡Intraoperative gallbladder assessment by the surgeon. CD, Clavien–Dindo; IQR, interquartile range; ABC™, achievable benchmark of care; ASA, American Society of Anesthesiologists physical status classification.

## Discussion

Based on data from a validated national registry, this study has developed benchmarks for ELC. Benchmarking against top peers has been found to be more efficient than comparison with average performance, particularly in settings with lower baseline performance^20^. This study established 10 benchmarks, aggregates of top-peer performance, using the ABC™ methodology^7,8^, allowing evaluation of routine clinical practice by identifying top-performing institutions within an unselected population. Together with the cohort’s percentile ranks, this provides a useful framework for facility self-audit.

The benchmarks are intended primarily as tools for quality improvement. Surgical facilities may compare local outcomes with benchmark values to identify potential performance gaps, initiate internal audit processes, or stimulate peer discussion regarding operative strategy, perioperative pathways, and resource utilization. Importantly, benchmarks are not prescriptive targets, as both case-mixes and local conditions may vary, but aim to support reflective practice and continuous improvement.

The outcomes of the present cohort regarding rates of conversion to open surgery, postoperative complications, reoperation, and readmission are comparable to those reported in previous international studies. These similarities support the external validity of the findings. The recent US large-scale ROBOCOP study^21^ reported an ELC conversion rate of 1.2%, and a 30-day readmission rate of 3.3%. Lucocq *et al*.^3^ observed a postoperative ELC complication rate of 4.9% in the UK, compared with 5.1% in the present study. The CholeS Study Group^4^ in UK and Ireland reported an ELC reoperation rate of 0.6%, compared with 0.7% here. By translating population outcomes into benchmark values, the present study extends existing literature from descriptive epidemiology towards aspirational reference standards.

The severe postoperative complications outcome was a constructed variable, with strict adjustments for missing data, excluding several centres from benchmark contribution to assure its validity. The benchmark for severe postoperative complications was 1.0%, as was the 75th percentile rank of the overall cohort, suggesting that it is attainable for many. Conversion to open surgery was uncommon but varied substantially between facilities, as did intraoperative drain placement, possibly indicating differences in operative decision-making. Variation was also observed in postoperative bile leakage, although this outcome was rare. Differences in reporting practices may have contributed, as some facilities may only have reported significant leakages. Injury to the common bile duct was not benchmarked individually owing to its scarcity. The observed inter-facility variation supports the rationale for benchmarking approaches, as average outcome reporting may conceal differences in practice patterns and operative decision-making.

Median LOS and its benchmark were both 0 days, indicating that ambulatory cholecystectomy is common in Sweden. Geographic factors likely contribute to variations in LOS, as certain regions in Sweden require overnight stay due to substantial travel distances. Operative duration was comparable to that reported in other studies^3,21^, despite routine use of IOC. Whereas the safety benefit of IOC in ELC remains a subject of debate, these findings suggest that operative time is not significantly increased when IOC is standardized.

ASA grade ≥ III and previous intraabdominal surgery were identified as independent preoperative risk factors for severe postoperative complications. An intraoperative assessment of an altered gallbladder morphology, an indirect sign of operative complexity, was common despite elective surgical indication and was also independently associated with severe postoperative complications. This association should be interpreted cautiously, as intraoperative assessment may be biased by surgeon subjectivity. Nevertheless, adjusted benchmark values remained low in these subgroups, indicating that favourable outcomes are achievable even in moderately complex ELC cases. Complementary subgroup analyses restricted to low-risk populations yielded slightly lower benchmark values, useful for performance assessment in these cohorts. Overall, the findings suggest that the original benchmark remain broadly applicable for assessment of institutional performance despite variation in patient risk and operative complexity.

Several methodological limitations should be acknowledged. First, benchmarking results depend on the quality and completeness of registry data. Although the GallRiks registry has demonstrated high validity and national coverage, residual registration bias cannot be excluded. To mitigate this, facilities with incomplete data were excluded from contributing to benchmark derivation, particularly affecting the benchmark for severe complications. Second, the data reflect practice within a single country, potentially limiting generalizability. Surgical facilities operating under different conditions may therefore interpret benchmarks, such as LOS, according to their circumstances. Third, the cohort included patients undergoing ELC for biliary colic or polyps, as well as delayed ELC following previous inflammation, reflecting real-world practice but introducing variation in case-mix between facilities. Multivariable logistic regression did not identify delayed ELC as an independent risk factor for severe postoperative complications, supporting the generalizability of the findings. Finally, the exclusion of patients with previous upper abdominal surgery and abnormal IOC aimed to reduce procedural heterogeneity but introduces selection bias and limit transferability to more complex cholecystectomy populations. An additional remark is that the ABC™ methodology identifies achievable outcome levels independent of causal effects, and therefore possible residual case-mix differences must be considered when interpreting the outcomes.

In conclusion, the present study established clinically relevant benchmarks for ELC in patients without intraoperative suspicion of bile duct obstruction. Together with the cohort’s percentile ranks, these benchmarks provide reference values for quality assessment, whereas the selection of specific performance indicators and implementation of improvement strategies remain at the discretion of individual facilities and surgeons.

## Supplementary Material

zrag175_Supplementary_Data

## Data Availability

The data sets generated and/or analysed during the present study are not publicly available but are available from the corresponding author on reasonable request.
