# Supplementary material for: Elective cholecystectomy benchmarks: nationwide registry study
Source: BJS Open. 2026 Oct 13;10(5):zrag175. doi: 10.1093/bjsopen/zrag175 (PMC13660693; doi:10.1093/bjsopen/zrag175)
Supplement: Supplementary file 1 [file zrag175_supplementary_data.docx]

Elective Cholecystectomy Benchmarks: Nationwide Registry Study

Ebba Kihlstedt Pasquier, MD^1,2^; Ellen Andersson, MD, PhD^1,3^; Johanna Österberg, MD, PhD^,4,5^; Per Sandström, MD, PhD^1,2^, Bergthor Björnsson, MD, PhD^1,2^

^1^ Department of Biomedical and Clinical Sciences, Linköping University, Linköping, Sweden

^2^ Clinical Department of Surgery in Linköping, Region Östergötland, Linköping, Sweden

^3^ Clinical Department of Surgery in Norrköping, Region Östergötland, Norrköping, Sweden

^4^ Department of Clinical Science and Education, Södersjukhuset, Karolinska Institutet, Stockholm, Sweden

^5^ Department of Surgery, Mora Lasarett, Mora, Sweden

**Corresponding author**

Ebba Kihlstedt Pasquier

Department of Surgery, Linkoping University Hospital, 581 85 Linkoping, Sweden

ORCID ID: 0000-0002-5754-3271

E-mail: [ebba.kihlstedt.pasquier@liu.se](mailto:ebba.kihlstedt.pasquier@liu.se)

**Supplementary Materials - Index**

| **Supplementary Methods** |  |
| --- | --- |
| Supplementary Table S1. Calculation of adjusted performance fraction, APF | *page 2* |
| Supplementary Table S2. Benchmark calculation, example | *page 2* |
| **Supplementary Results** |  |
| Supplementary Table S3. Baseline characteristics of the study population | *page 3* |
| Supplementary Table S4. Intraoperative variables | *page 4* |
| **Supplementary Appendixes** |  |
| Supplementary Appendix 1. STROBE Statement—Checklist  Supplementary Appendix 2. Clavien-Dindo (CD) classification | *page 5*  *page 7* |
| Supplementary Appendix 3. Questionnaire completed by a surgical center with excellent results | *page 8* |
| **Supplementary Figures** |  |
| Supplementary Figure S1. Facilities' surgical volume and ASA classification | *page 10* |
| Supplementary Figure S2. Benchmark cohorts and ASA classification | *page 10* |
|  |  |
|  |  |

**Supplementary Methods**

| **Supplementary** Table S1. Calculation of adjusted performance fraction, APF | | | |
| --- | --- | --- | --- |
| Hospital | Procedure, n | Severe complication,  Clavien-Dindo ≥ III | APF =  (events+1)/(surgeries+2) |
| A | 306 | 10 | 0,035714 |
| B | 175 | 1 | 0,011299 |
| C | 5 | 0 | 0,142857 |
| D | 384 | 12 | 0,033679 |
| E | 749 | 19 | 0,026631 |
| F | 213 | 0 | 0,004651 |
| G | 675 | 19 | 0,029542 |
| H | 737 | 15 | 0,021651 |
| I | 640 | 13 | 0,021807 |
| J | 265 | 1 | 0,007491 |
| K | 1489 | 31 | 0,021462 |
| L | 1086 | 13 | 0,012868 |
| M | 606 | 13 | 0,023026 |

| **Supplementary** Table S2. Benchmark calculation, example | | | | | | |
| --- | --- | --- | --- | --- | --- | --- |
| Hospital | Procedure, n | | Severe complication,  Clavien-Dindo ≥ III | Missing data in specific variable | Missing data in overall complication registration | **APF ranking** |
| F | 213 | 0 | | 33,33% | 0,47% | 0,004651 |
| J | 265 | 1 | | 0,00% | 7,17% | 0,007491 |
| B | 175 | 1 | | 0,00% | 0,57% | 0,011299 |
| L | 1086 | 13 | | 25,81% | 0,28% | 0,012868 |
| K | 1489 | 31 | | 1,83% | 0,07% | 0,021462 |
| H | 737 | 15 | | 0,00% | 2,85% | 0,021651 |
| I | 640 | 13 | | 9,76% | 0,63% | 0,021807 |
| M | 606 | 13 | | 18,52% | 0,00% | 0,023026 |
| E | 749 | 19 | | 3,03% | 1,20% | 0,026631 |
| G | 675 | 19 | | 0,00% | 0,15% | 0,029542 |
| D | 384 | 12 | | 0,00% | 0,26% | 0,033679 |
| A | 306 | 10 | | 0,00% | 0,00% | 0,035714 |
| C | 5 | 0 | | 0,00% | 0,00% | 0,142857 |
| **Total cohort: 7 330 patients** | | | |  |  |  |
| **Ten percent: 733 patients** | | | |  |  |  |
| Surgical facilities marked in yellow are excluded from benchmark contribution due to zero events or ≥ 5% missing data in overall complication registration or ≥ 5% missing data in registration of specific variable (consequence/treatment). | | | | | | |
| Benchmark of top performing surgical facilities accounting for at least 10% of all procedures:  (1+31) / (175+1489) = 0.01923 or 1.923% | | | | | | |

**Supplementary Results**

## **Supplementary Table S3**. Baseline characteristics of the study population, cohort n = 48 564

|  | **Median** | **IQR** | **Missing nr (%)** |
| --- | --- | --- | --- |
| Age at time of surgery (years) | 50.0 | 38.0 - 63.0 | 0 |
| Weight (kg) | 80.0 | 70.0 - 91.0 | 4 457 (9.2) |
| Height (cm) | 168.0 | 163.0 - 175.0 | 4 519 (9.3) |
| BMI (kg/m2) | 27.8 | 24.8 - 31.3 | 4 614 (9.5) |
|  | **Number** | **Percent** | **Missing nr (%)** |
| Sex ratio (M:F) | 14 926 : 33 637 | 30.7 : 69.3 | 1 (0.0) |
| Surgical indication |  |  | 0 |
| Biliary colic | 36 337 | 74.8 | NA |
| Gallstone related complication^a^ | 11 677 | 24.0 | NA |
| Previous cholecystitis | 6 405 | 13.1 | NA |
| Previous cholecystitis and pancreatitis | 609 | 1.3 | NA |
| Previous pancreatitis | 3 048 | 6.3 | NA |
| Previous clinical icterus, total bilirubin>50 µmol/L or gallstone in CBD, *without concurrent cholecystitis or pancreatitis* | 1 307 | 2.7 | NA |
| Unclassified gallstone complication^a^ | 308 | 0.6 | NA |
| Gallbladder polyp | 550 | 1.1 | NA |
| Comorbidity^b^ | 10 375 | 26.2 | 215 (0.5) |
| Cardiovascular disease | 6 306 | 15.9 | 262 (0.7) |
| Diabetes^c^ | 2 401 | 6.1 | 248 (0.6) |
| Asthma/COPD | 2 845 | 7.2 | 278 (0.7) |
| Increased risk of bleeding^d^ | 1 519 | 3.8 | 311 (0.8) |
| Immunosuppression^e^ | 723 | 1.8 | 307 (0.8) |
| Smoking | 2 232 | 5.6 | 2 741 (6.9) |
| Prior abdominal procedure^b^ | 759 | 1.9 | 0 |
| ASA classification |  |  | 0 |
| ASA I | 20 907 | 43.1 | NA |
| ASA II | 23 441 | 48.3 | NA |
| ASA III | 4 166 | 8.6 | NA |
| ASA IV | 50 | 0.1 | NA |
| ASA V | 0 | 0 | NA |
| ^a^ Previous cholecystitis, pancreatitis or common bile duct stone/elevated bilirubin.  ^b^ The term encompass previous abdominal procedures not already excluded, including pre-existing conditions, or anatomical variations considered to affect or complicate the operation.  Variables introduced in GallRiks on the 1^st^ of September 2016. Cohort size: 39 633 patients.  ^c^ Lifestyle- or pharmacologically treated diabetes.  ^d^ von Willebrand disease, hemophilia, ongoing anticoagulant therapy, or INR > 1.5.  ^e^ Current immunosuppressive therapy.  BMI, body mass index. CBD, common bile duct. COPD, chronic obstructive pulmonary disease. ASA, American Society of Anesthesiologists physical status classification. NA, not applicable. INR, International Normalized Ratio. | | | |

**S**upplementary Table S4. Intraoperative variables, cohort n = 48 564

|  | **Number (%)** | **Missing (%)** |
| --- | --- | --- |
| Antibiotics | 5 108 (10.5) | 0 |
| Thromboprophylaxis | 9 704 (20.0) | 0 |
| Laparoscopic access |  | 1 (0.0) |
| Open, near umbilicus | 44 772 (92.2) | NA |
| Verres needle | 2 983 (6.1) | NA |
| Other technique | 808 (1.7) | NA |
| Dissection of gallbladder |  | 25 (0.1) |
| Infundibulum first | 45 255 (93.2) | NA |
| Fundus first | 2 987 (6.2) | NA |
| No dissection before conversion | 297 (0.6) | NA |
| Closure of cystic artery specified |  | 4 852 (10.0) |
| 1 clip | 3 673 (8.4) | NA |
| 2 clips | 31 968 (73.1) | NA |
| Diathermia | 4 340 (9.9) | NA |
| Other (including open techniques) | 1 664 (3.8) | NA |
| Artery not detected | 2 067 (4.7) | NA |
| Closure of cystic duct specified |  | 4 852 (10.0) |
| 1 clip | 718 (1.6) | NA |
| ≥2 clips | 42 172 (96.5) | NA |
| Other (including open techniques) | 797 (1.8) | NA |
| Cystic duct not seen | 25 (0.1) | NA |
| Abdominal drainage | 1 578 (3.2) | 0 |
| Laparoscopic subtotal cholecystectomy | 24 (0.0) | 0 |
| Conversion to open surgery | 653 (1.3) | 0 |
| Anatomy related ^a^ | 556 (85.1) | NA |
| Bleeding | 46 (7.0) | NA |
| Suspected bile duct injury | 16 (2.5) | NA |
| Other complication | 35 (5.4) | NA |
| Adverse event | 15 044 (31.0) | 0 |
| Accidental gallbladder perforation | 14 797 (30.5) | 24 (0.0) |
| Intestinal perforation | 57 (0.1) | 0 |
| Bile duct injury | 61 (0.1) | 0 |
| *< 1/3 of the circumference* | 25 (41.0) | NA |
| *> 1/3 of the circumference* | 4 (6.6) | NA |
| *Resection of essential part of bile duct wall* | 2 (3.3) | NA |
| *Resection of entire circumference* | 2 (3.3) | NA |
| *Other bile leakage (e.g. subvesical bile duct)* | 28 (45.9) | NA |
| Bleeding ^b^ | 170 (0.4) | 0 |
| Other complication ^c^ | 185 (0.4) | free text |
| Macroscopic assessment of gallbladder |  | 0 |
| Normal gallbladder (745 with polyps) | 34 144 (70.3) | NA |
| Cholecystitis (acute or chronic) | 14 034 (28.9) | NA |
| Suspected malignancy | 87 (0.2) | NA |
| Other finding | 299 (0.6) | NA |
| ^a^ “Anatomy related” includes adhesions, advanced cholecystitis and unclear anatomy.  ^b^ Bleeding that requires transfusion or conversion to open surgery.  ^c^ Other complications includes cardiovascular and pulmonary complications, medicotechnical issues (problems with equipment that affect the outcome, e.g. prolongation of operation time due to camera replacement), use of hemostatic products to control bleeding and suturing of intestinal damage without perforation.  NA, not applicable. | | |

**Supplementary Appendixes**

**Supplementary Appendix 1.**

STROBE Statement—Checklist of items that should be included in reports of ***cohort studies***

|  | | Item No | Recommendation | Page No |
| --- | --- | --- | --- | --- |
| **Title and abstract** | | 1 | (*a*) Indicate the study’s design with a commonly used term in the title or the abstract | 1 |
|  |  |  | (*b*) Provide in the abstract an informative and balanced summary of what was done and what was found | 2-3 |
| Introduction | | | | |
| Background/rationale | | 2 | Explain the scientific background and rationale for the investigation being reported | 4 |
| Objectives | | 3 | State specific objectives, including any prespecified hypotheses | 4 |
| Methods | | | | |
| Study design | | 4 | Present key elements of study design early in the paper | 5 |
| Setting | | 5 | Describe the setting, locations, and relevant dates, including periods of recruitment, exposure, follow-up, and data collection | 5 |
| Participants | | 6 | Give the eligibility criteria, and the sources and methods of selection of participants. Describe methods of follow-up | 5 |
| Variables | | 7 | Clearly define all outcomes, exposures, predictors, potential confounders, and effect modifiers. Give diagnostic criteria, if applicable | 7-9 |
| Data sources/ measurement | | 8* | For each variable of interest, give sources of data and details of methods of assessment (measurement). Describe comparability of assessment methods if there is more than one group | 5-7, 9 |
| Bias | | 9 | Describe any efforts to address potential sources of bias | 9 |
| Study size | | 10 | Explain how the study size was arrived at | 5 |
| Quantitative variables | | 11 | Explain how quantitative variables were handled in the analyses. If applicable, describe which groupings were chosen and why | 9 |
| Statistical methods | | 12 | (*a*) Describe all statistical methods, including those used to control for confounding | 9 |
|  |  |  | (*b*) Describe any methods used to examine subgroups and interactions |  |
|  |  |  | (*c*) Explain how missing data were addressed |  |
|  |  |  | (*d*) If applicable, explain how loss to follow-up was addressed |  |
|  |  |  | (*e*) Describe any sensitivity analyses |  |
| Results | | | |  |
| Participants | | 13* | (a) Report numbers of individuals at each stage of study—eg numbers potentially eligible, examined for eligibility, confirmed eligible, included in the study, completing follow-up, and analysed | 10  Figure 1 |
|  |  |  | (b) Give reasons for non-participation at each stage |  |
|  |  |  | (c) Consider use of a flow diagram |  |
| Descriptive data | | 14* | (a) Give characteristics of study participants (eg demographic, clinical, social) and information on exposures and potential confounders | 10  Table S1 |
|  |  |  | (b) Indicate number of participants with missing data for each variable of interest |  |
|  |  |  | (c) Summarise follow-up time (eg, average and total amount) |  |
| Outcome data | | 15* | Report numbers of outcome events or summary measures over time | 10-13  Table 1  Table S2-3 |
| Main results | 16 | (*a*) Give unadjusted estimates and, if applicable, confounder-adjusted estimates and their precision (eg, 95% confidence interval). Make clear which confounders were adjusted for and why they were included | | 12  Figure 2  Table 2-3 |
|  |  | (*b*) Report category boundaries when continuous variables were categorized | |  |
|  |  | (*c*) If relevant, consider translating estimates of relative risk into absolute risk for a meaningful time period | |  |
| Other analyses | 17 | Report other analyses done—eg analyses of subgroups and interactions, and sensitivity analyses | | 13  Figure S1-2 |
| Discussion | | | | |
| Key results | 18 | Summarise key results with reference to study objectives | | 13-16 |
| Limitations | 19 | Discuss limitations of the study, taking into account sources of potential bias or imprecision. Discuss both direction and magnitude of any potential bias | | 15-16 |
| Interpretation | 20 | Give a cautious overall interpretation of results considering objectives, limitations, multiplicity of analyses, results from similar studies, and other relevant evidence | | 16 |
| Generalisability | 21 | Discuss the generalisability (external validity) of the study results | | 14 |
| Other information | | | | |
| Funding | 22 | Give the source of funding and the role of the funders for the present study and, if applicable, for the original study on which the present article is based | | 1 |

*Give information separately for exposed and unexposed groups.

**Note:** An Explanation and Elaboration article discusses each checklist item and gives methodological background and published examples of transparent reporting. The STROBE checklist is best used in conjunction with this article (freely available on the Web sites of PLoS Medicine at http://www.plosmedicine.org/, Annals of Internal Medicine at http://www.annals.org/, and Epidemiology at http://www.epidem.com/). Information on the STROBE Initiative is available at http://www.strobe-statement.org.

**Supplementary Appendix 2**

**Clavien-Dindo (CD) classification**

- Existing CD values when available (variable introduced in 2021)
- Antibiotics or blood transfusion = CD 2
- ERCP, PTC or percutaneous drainage = CD 3a
- Reoperation = CD 3b
- Care in ICU = CD 4
- Death within 30 days of surgery = CD 5
- Classification of complications reported as free text:

| CD 1 | - Pain, due to likely analgesic treatment - Urinary retention, catheterization, urinary catheter - Unspecified bleeding (no reoperation, transfusion or drainage) - Pancreatitis or fever, due to likely analgesic or antipyretic treatment - Bile leakage without specification, or a fluid collection reported on computed tomography |
| --- | --- |
| CD 2 | - Thrombosis/embolism (presumed deep venous thrombosis), due to likely medical treatment - Pulmonary embolism, myocardial infarction, stroke, due to likely medical treatment - Paralytic or mechanical ileus, due to likely nasogastric decompression - Antibiotic treatment or likely antibiotic treatment   - Abscess (no percutaneous drainage specified)   - Sepsis (no organ failure or ICU care specified)   - Superficial wound infection, urinary tract infection, pneumonia |
| CD 3a | - Bile duct obstruction, due to likely ERCP treatment - Wound dehiscence without specified treatment, classified as CD 3a due to likely resuturing under minimum local anesthesia |
| CD 3b | - Reoperation or likely reoperation   - Intestinal injury without specified treatment. If both reoperation and percutaneous drainage were registered as "No", the complication was instead classified as CD 2, due to likely antibiotic treatment.   - Anastomotic leakage after intestinal resection   - Port-site hernia   - Adhesive small bowel obstruction |
| CD 4a | Care in ICU |
| CD 5 | Death within 30 days of surgery |

**Supplementary Appendix 3**

**Q**uestionnaire completed by a surgical center with excellent results

**Elective laparoscopic cholecystectomy case-mix (GallRiks data)**

| **Indicators** | **Center of excellence** | **Swedish cohort** |
| --- | --- | --- |
| Patient selection: ASA I-II | 89.5 | median 91.8 (IQR 87.9-96.8) |
| Indication: biliary colic or polyp | 80.3 | median 77.4 (IQR 67.9-85.8) |
| Intraoperative inflammation | 28.0 | median 27.6 (IQR 22.7-33.9) |

All values are expressed as percentages.

**Nr 1 Question:**

What do you think you do differently from other hospitals when performing laparoscopic cholecystectomy?

Answer:

**Preoperative decision-making**

- Do you apply specific criteria for **delaying surgery after acute cholecystitis?**	Yes ☒ No ☐

Which:

How long is the delay after acute cholecystitis?	(2-3 months)

- Do you routinely review cases in **preoperative conferences**?	Yes ☐ No ☒
- Are difficult cases **pre-identified and scheduled with senior surgeons**?	Yes ☒ No ☐
- Do you **refer** potentially difficult cases to another hospital?	Yes ☒ No ☐

**2. Preoperative optimization**

- Is there a **standard preoperative work-up protocol**?	Yes ☐ No ☒

Which:

- Are patients with prior inflammation treated with **antibiotic prophylaxis**?	Yes ☐ No ☒
- Do you routinely perform **preoperative imaging**?	Yes ☐ No ☒

**3. Surgeon experience and training**

- **How many surgeons** in your department perform LC?
- Are **resident surgeons** allowed to perform ELC cases **independently**?	Yes ☒ No ☐
- Are **consultants present** during surgery?	Yes ☐ No ☒
- Do you have **high-volume LC surgeons** (>50/year), how many?	Yes ☐ No ☒
- Are there usually **one or two surgeons** scrubbed in during LC?	1 ☐ 2 ☒
- Are surgeons encouraged to **ask for help intraoperatively**?	Yes ☒ No ☐
- Do you have **video review** / **morbidity conferences** of complications?	Yes ☒ No ☐

**4. Intraoperative technique**

- Do you follow a **standard dissection sequence**?	Yes ☒ No ☐

Which:

- Are **energy devices** standardized?	Yes ☐ No ☒
- Is **critical view of safety mandatory** before clipping?	Yes ☒ No ☐
- Do you routinely document the surgery or critical view **(photo or video)**?	Yes ☒ No ☐
- Do you have a protocol when **anatomy is unclear**?	Yes ☐ No ☒

Which:

- Do you apply a **routine or** **time limit** before asking for **help intraoperatively?**	Yes ☐ No ☒

If specific dissection moment, which:

If specific operative duration, which:

- Are surgeons encouraged to **bail out early** if dissection is difficult**?**	Yes ☒ No ☐

**6. Team and operating room processes**

- Are the **staff** regularly assigned to cholecystectomy cases?	Yes ☐ No ☒
- Do you use **standardized instrument sets**?	Yes ☒ No ☐
- Are there **intraoperative checklist points specific to ELC**?	Yes ☐ No ☒

Which:

**7. Postoperative management - early detection of complications and management**

- Patients routinely **discharged** the same day (SD) or stay overnight (ON)?	SD ☒ ON ☐
- Do you have a **standardized postoperative follow-up**?	Yes ☐ No ☒

What does it look like? How long after surgery? By which profession? Phone call or visit?

**Supplementary Figures**

**S**upplementary Figure S1. Facilities' surgical volume and ASA classification

ASA, American Society of Anesthesiologists physical status classification (ASA).

Supplementary Figure S2. Benchmark cohorts and ASA classification

ASA, American Society of Anesthesiologists physical status classification. The numbers in parentheses refer to number of facilities contributing to the benchmark. *Readmission was introduced in the registry on the 1^st^ of September 2016. Cohort size: 39 633 patients.
